# Interbreeding area movement of an adult humpback whale between the east Pacific Ocean and southwest Indian Ocean

**DOI:** 10.1098/rsos.241361

**Published:** 2024-12-11

**Authors:** Ekaterina Kalashnikova, Natalia Botero-Acosta, Esteban Duque Mesa, Mar Palanca Gascón, Patrick Lyne, Ted Cheeseman, Alex Vogel, Amy Kennedy, Aylin Akkaya

**Affiliations:** ^1^ Tanzania Cetaceans Program, Zanzibar, Tanzania; ^2^ Bazaruto Center for Scientific Studies (BCSS), Benguerra, Mozambique; ^3^ Macuáticos Colombia Foundation. Carrera, Medellín 86 #30A-46, Colombia; ^4^ Jardín Botánico del Pacífico Mecana, Chocó, Colombia; ^5^ Madre Agua Colombia, Sabaneta, Antioquia, Colombia; ^6^ DMAD-Marine Mammals Research Association Erenkoy, Istanbul, Turkey; ^7^ Marine Ecology Research Centre, Southern Cross University, Lismore, Australia; ^8^ Happywhale, Santa Cruz, CA, USA; ^9^ Cooperative Institute for Climate, Ocean, and Ecosystem Studies (CICOES), University of Washington, Seattle WA, USA

**Keywords:** humpback whales, breeding grounds, eastern Pacific Ocean, southwest Indian Ocean, migration, photo-identification

## Abstract

Humpback whales undertake one of the longest known migrations of any mammal. While their migration route generally extends between latitudes, the breeding stocks are longitudinally separated and display high site fidelity to their feeding grounds. While there is an indication of certain breeding stocks overlapping with each other, the current information on the migration routes of humpback whales within the Southern Hemisphere limits our understanding of the extent of this exchange. Presented here is the longest documented great-circle distance between sightings on wintering grounds of two different ocean basins of an adult male humpback whale, involving two breeding stocks in the eastern Pacific (stock G) and southwest Indian Ocean (stock C). These two stocks are separated by a minimum of 120° longitude, and a great-circle distance of 13 046 km. This extreme distance movement demonstrates behavioural plasticity, which may play an important role in adaptation strategies to global environmental changes and perhaps be an evolved response to various pressures, underlining the importance of consolidation of global datasets on wide-ranging marine mammals.

## 1. Introduction

Migratory behaviour can be observed in diverse taxonomic groups and serves a crucial role in the lifespan of an animal. Feeding and breeding grounds may be substantially separated. Often, this corresponds with high site fidelity and stable migration routes [1]. Connectivity and isolation are among the factors that shape the population structure evolution [2]. Humpback whales (*Megaptera novaeangliae*) undertake one of the longest known seasonal migrations of all mammals [3]. This globally distributed species typically displays annual migration between low-latitude breeding grounds and high-latitude feeding grounds [4]. The migratory destinations have been shown to remain consistent [5], with timing being remarkably similar over many years [6]. While their latitudinal migration route between feeding and breeding grounds is known to exceed 8000 km in a single direction [1,3,7–11], the longitudinal movement is defined as atypical for this species [12].

According to the International Whaling Commission, seven breeding stocks of humpback whales are recognized in the Southern Hemisphere (A–G). East Africa encompasses breeding stock C, comprising sub-stock C1 along the East African coast, with its southern extent including the coasts of South Africa, Mozambique and southern Tanzania C1(S), and its northern extent covering the coasts of central and northern Tanzania and Kenya C1(N); C2 along the central Mozambique Channel Islands, C3 along the Madagascar Ridge and C4 around the Mascarene islands (La Reunion, Mauritius and Rodrigues; [5]; figure 2). The interoceanic exchange between the breeding stocks has been reported with mixed gene flows between the western (A), and eastern (B1, B2) breeding stocks in the South Atlantic and Indian Ocean (C1) [13,14]. Genetic and photo-identification studies indicated that there is a considerable level of connectivity between C2 and C3 sub-regions [15–17]. On the contrary, both C2 and C3 stocks reveal a low probability of exchange with C1. However, intra-seasonal movement between C3 and the northern extent of C1 has been revealed by satellite tagging studies [18]. These breeding stocks are known to be feeding in Antarctica with few supplementary feeding events documented in low latitudes [19].

Recent studies demonstrate deviation from the known migratory destinations as well as interocean longitudinal movement patterns [11,13,14,20]. For instance, a female humpback whale was photographed in the breeding ground off Ecuador in 1996 and later photo-identified in the non-adjacent breeding ground off Brazil in 1998, representing discrete stocks G and A, and an estimated distance of 12 000 km and with a separation of more than 40° longitude [20]. A different female was seen in Brazil (1999) and Madagascar (2001), which are separated by a minimum of 9800 km [13]. Similarly, a comprehensive study reported the matching of six individuals between breeding grounds in the Abrolhos Bank (Brazil) and Cape Town (South Africa) between 2002 and 2021 [14]. Another subadult male was first photographed off Madagascar in 2000 and later seen alone in 2002 in Gabon [11]. One of the longest great-circle distances (11 261 km) between humpback whale sightings—the Mariana Islands and Mexico—with one year in between has been documented [9] demonstrating 108.6° longitudinal separation. The longest known recorded migration to date with the greatest longitudinal distance of 143° between sightings was travelled by a female humpback whale between feeding and breeding areas [12]. These rare resightings provide certain insights into the interoceanic movement occasions of humpback whales [9,11–13,20].

Photo-ID and genetic studies have advanced the understanding of movement patterns within and among the breeding stocks and their migration routes [2,21–23]. Therefore, the current study employed the Happywhale platform (https://happywhale.com/) to assess the potential resightings of humpback whales between Zanzibar and other regions. This study presents the furthest documented to date great-circle distance between sightings of an adult male humpback whale on two breeding grounds of the eastern South Pacific and the southwest Indian Ocean and underlines the importance of transboundary research effort and citizen science to understand potential drivers and population impact of interoceanic movements of humpback whales.

## 2. Material and methods

Dedicated vessel-based surveys have been conducted since 2010 (Macuáticos Colombia Foundation) and 2017 (Madre Agua Colombia) in Colombia and since 2020 in Zanzibar (Tanzania Cetaceans Program initiative). Surveys were conducted throughout the breeding season (July to September) in Zanzibar and from July to October in Colombia. Location, acoustic behaviour, group type, group size, spatial distribution and photo-identification images were collected in all locations. Fluke images were uploaded to Happywhale.com a web-based cetacean photo-ID platform. The platform contains photo-ID-based encounter data of over 91 000 individually identified humpback whales in over 335 000 encounters, including 103 individuals sighted in Tanzania and 1168 individuals in Colombia, as of March 2024. Fluke images from Zanzibar and Colombia were initially matched with the global dataset of Happywhale by automated image recognition and were then confirmed or rejected by trained Happywhale data managers. Fluke images with no confirmed match found were screened for image quality and added to the reference dataset as new individuals with IDs, following the Happywhale data management methods [21].

The geographic locations of matches were plotted in QGIS software (v. 3.36.0). The method described in Bowditch [24] was used to identify the shortest distance between two sightings—the great-circle distance—considering the spherical surface of the earth. The potential migratory route, including the nearest foraging area around South Georgia and South Shetland, was also estimated, and the two distances—the Colombia feeding area and the Tanzania feeding area—were calculated accordingly. The South Georgia and South Shetland area was chosen as a potential foraging stop for the calculation of distances, as it is known to be a feeding ground for stock G [23,25,26].

## 3. Results

**Figure 1.**
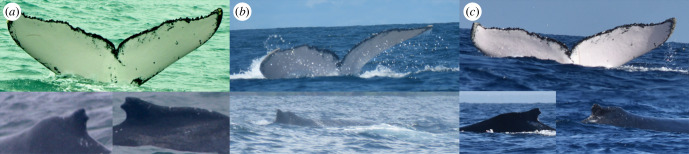
(*a*) Humpback whale (*Megaptera novaeangliae*), observed in the Gulf of Tribugá, northern Colombian Pacific, on 10 July 2013. Photographed by N. Botero-Acosta of Fundación Macuáticos Colombia. *(b)* Humpback whale (*Megaptera novaeangliae*), observed in Bahía Solano, northern Colombian Pacific, on 13 August 2017. Photographed by E. D. Mesa of Madre Agua Colombia. *(c)* Humpback whale (*Megaptera novaeangliae*), observed in Zanzibar channel, off Fumba on 22 August 2022. Photographed by E. Kalashnikova.

One humpback whale, which appeared to be an adult based on the behaviour and size estimation, was first photographed off the Gulf of Tribugá, northern Colombian Pacific, on 10 July 2013 (figure 1*a*). This adult was part of a competitive group that included seven humpbacks and was associated with a group of bottlenose dolphins (*Tursiops truncatus*). The same individual was resighted five years later (13 August 2017) in Bahía Solano, roughly 78 km from the 2013 sighting location (figure 1*b*). This whale was again within a competitive group of eight individuals, which included a mother and calf pair. These sightings were recorded in Happywhale (https://happywhale.com/individual/13284). The most recent sighting took place off Fumba in the Zanzibar channel, southwest Indian Ocean, on 22 August 2022 (figure 1*c*). The individual was again in a competitive group of five whales. A Happywhale record for this encounter is presented at: https://happywhale.com/individual/13284;enc=356519. The whale’s competitive-group behaviour and a photograph showing a lack of a hemispherical lobe [27] near the genital slit taken in Colombia in 2013 suggest that this was a male humpback whale.

**Figure 2.**
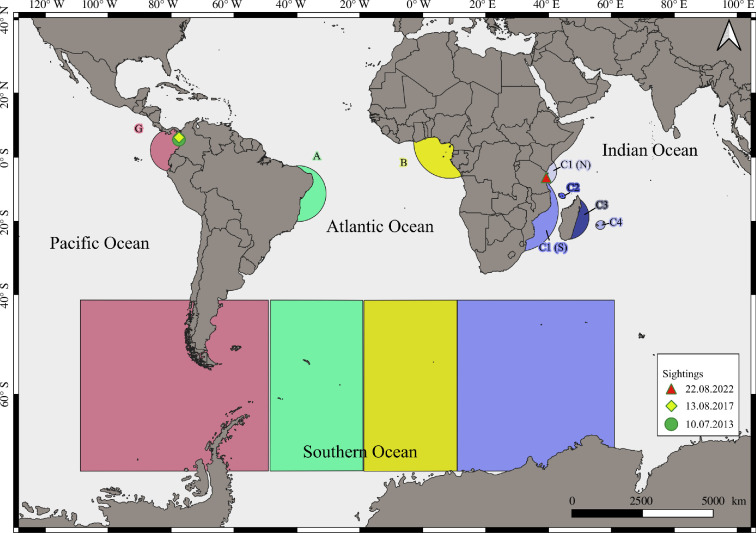
The sighting locations of the male humpback whale between the breeding grounds G and C. Squares represent primary feeding grounds of the Southern Hemisphere humpback whale breeding stocks (BS) G, A, B and C, with the latter split into C1 southern (C1S) and C1 northern (C1N) extents, C2, C3 and C4. Semicircles represent their respective core breeding grounds (adapted from [19]).

The exact migration route for this individual is unknown and may or may not embrace multiple latitudinal migrations between breeding grounds in Colombia and feeding grounds in the Western Antarctic Peninsula (WAP), as well as plausible movements from stock G to stocks A and B, using feeding grounds associated with these stocks, prior to arrival at the Zanzibar breeding ground. Sightings in Colombia in 2013 and Zanzibar in 2022 are separated by 13 046 km great-circle distance and 120° of longitude. This represents the longest recorded great-circle distance between sightings on two breeding grounds of a photo-identified adult male humpback whale, which is the first record of a humpback whale alternating breeding grounds between the Pacific and Indian Oceans (figure 2).

## 4. Discussion

Humpback whales often display strong site fidelity to specific breeding grounds, but with notable exceptions. Breeding ground changes have been known to occur [13,20,26–31], but the full extent of these shifts is unknown. It is known that mammals’ dispersal is often sex biased [32]. Earlier studies demonstrated that males tend to travel more between the breeding grounds driven by a male dominance polygyny mating system, characteristic of humpback whales [4,18,29]. At the same time, recent studies documented females undertaking substantial movements between different breeding grounds, despite their demonstrated high site fidelity to particular breeding areas [12,13,20]. Therefore, it is apparent that loyalty to the original breeding grounds may not necessarily be gender related. The long-distance travel described here fits within the male-biased dispersal scheme defined by Greenwood [33]. Interestingly, the same author suggested that this tendency is more typical for young animals. It cannot be precisely determined when the breeding area shift happened for this whale, but it can be said that the individual appears to have been a sexually mature male when first sighted in 2013 and during the movement between the east Pacific and western Indian Ocean after August 2017. When this male was seen in Zanzibar in 2022, assuming sexual maturity at a minimum of 6 years of age, he was at least 15 years old. The long-distance movement presented here appears to be atypical and raises the question as to what its drivers are, which could include but not necessarily be limited to mating strategies.

Other reasons behind this unusual new habitat exploration may be global climatic changes and altered environmental conditions and events [34–36]. Krill distribution in the Southern Ocean fluctuates yearly [37], impacting humpback whale distribution on the feeding grounds [35,37,38], which may in turn lead to altering wintering destinations, ensuring energy budget optimization. On the other hand, population increases may also be a driver of these breeding ground shifts, when animals may need to explore new breeding and/or feeding areas due to competition from larger, more established males in both areas. The exact cause or drivers of these breeding habitat shifts can only be speculated due to the current limited data availability on humpback whale behavioural ecology.

Documenting unique cross-boundary interoceanic matches provides further evidence on the inter-seasonal and inter-areal movement ranges of humpback whales, demonstrating the complexity of migration routes and population dynamics as well as habitat use. With Zanzibar representing potentially overlapping areas between the northern extent of C1 [19] and a probable range expansion from C3 [18], it is unknown whether the individual from this report followed the C1 or C3 migratory stream, and hence what role Tanzanian waters play with regard to the migration of humpback whales. Subsequent regional matches (E Kalashnikova 2023, unpublished data), revealed most recently between whales sighted in Tanzanian waters and the broader western Indian Ocean (WIO), as well as South Georgia to Maputo match https://happywhale.com/individual/32364;enc=374725 (A Kennedy 2018, unpublished data), which linked East Africa breeding grounds with feeding areas, further support the hypothesis that Tanzania may play an important role in regional and global connectivity, seasonally hosting representatives from within and beyond the WIO region, enabling the gene flow essential for the recovering populations.

To get more insights into the migration ecology of the species that move on an ocean-wide or even possibly global scale, transboundary research effort and sustained collaborative long-term monitoring are needed. Understanding how frequently these breeding area shifts occur would help to assess the magnitude of population-level effects. Further studies investigating global genetic structure and photo-ID, regionally and globally, will help to better explain and understand this phenomenon.

## Data Availability

The datasets supporting this article have been uploaded to Dryad [39].
